# Real-time tracking of Tomato brown rugose fruit virus (ToBRFV) outbreaks in the Netherlands using Nextstrain

**DOI:** 10.1371/journal.pone.0234671

**Published:** 2020-10-08

**Authors:** Bart T. L. H. van de Vossenberg, Michael Visser, Maaike Bruinsma, Harrie M. S. Koenraadt, Marcel Westenberg, Marleen Botermans

**Affiliations:** 1 National Reference Centre of Plant Health, Dutch National Plant Protection Organization, Wageningen, The Netherlands; 2 Naktuinbouw, Roelofarendsveen, The Netherlands; Clemson University, UNITED STATES

## Abstract

Tomato brown rugose fruit virus (ToBRFV) is a *Tobamovirus* that was first observed in 2014 and 2015 on tomato plants in Israel and Jordan respectively. Since the first description, the virus has been reported from all continents except Oceania and Antarctica, and has been found infecting both tomato and pepper crops. In October 2019, the Dutch National Plant Protection Organization received a ToBRFV infected tomato sample as part of a generic survey targeting tomato pests. Presence of the virus was verified using Illumina sequencing. A follow-up survey was initiated to determine the extent of ToBRFV presence in the Dutch tomato horticulture and identify possible linkages between ToBRFV genotypes, companies and epidemiological traits. Nextstrain was used to visualize these potential connections. By November 2019, 68 companies had been visited of which 17 companies were found to be infected. The 50 ToBRFV genomes from these outbreak locations group in three main clusters, which are hypothesized to represent three original sources. No correlation was found between genotypes, companies and epidemiological traits, and the source(s) of the Dutch ToBRFV outbreak remain unknown. This paper describes a Nextstrain build containing ToBRFV genomes up to and including November 2019. Sharing data with this interactive online tool will enable the plant virology field to better understand and communicate the diversity and spread of this new virus. Organizations are invited to share data or materials for inclusion in the Nextstrain build, which can be accessed at https://nextstrain.nrcnvwa.nl/ToBRFV/20191231.

## Introduction

*Tomato brown rugose fruit virus* belongs to the genus *Tobamovirus*. The virus was first described by Salem *et al*. in 2016 after a finding in tomato (*Solanum lycopersicum*) plants in Jordan [1]. Symptoms appeared to be consistent with a virus that was causing problems in Israel since 2014, with tomato plants showing mosaic patterns on leaves, and occasionally narrowing of leaves and yellow spotted fruits. Pepper (*Capsicum* spp.) can also be affected by the virus, producing symptoms similar to those observed on tomato [2]. However, outbreaks in pepper have only been reported from Mexico, Jordan and Italy [2–4]. Several other plant species, including *Petunia* spp. and *Solanum nigrum* could be infected under laboratory conditions [5]. The tomato *Tm-2*^*2*^ *R*-gene, which confers resistance to tobacco mosaic virus (TMV) and tomato mosaic virus (ToMV), offers no protection against tomato brown rugose fruit virus (ToBRFV), hence the virus is regarded a major threat for tomato crops [1, 5]. As with other tobamoviruses, ToBRFV is stable and can easily be transmitted by contact (e.g. farming equipment, hands, clothing and plant-to-plant contact), and propagation material (grafts, cuttings) [6]. Seed transmission of ToBRFV is presumed [7], and bumblebees (*Bombus terrestris*) have been reported as vectors for ToBRFV and potentially contribute to the spread of the disease [8]. Following the initial findings in Jordan and Israel, the virus was reported from the State of Palestine [9], Mexico [10], the United States of America [11], Germany [12], and Italy [13] in 2018, and Turkey [14], China [15], the United Kingdom [16], the Netherlands [17], Greece [18], and Spain [19] in 2019. In 2020, ToBRFV outbreaks were reported from France [20] and Egypt [21].

ToBRFV has a similar genome organization to other tobamoviruses, and consists of a ~6.4 kb monopartite linear single stranded positive sense RNA (ssRNA) encoding four proteins: the small replicase subunit, a RNA-dependent RNA polymerase (RdRp) which is translated through a suppression of translation at the end of ORF1, a movement protein (MP), and a coat protein (CP) [1]. To date, nine (near) complete ToBRFV genome sequences have been deposited in NCBI Genbank (MK133095, MN167466, MK319944, KX619418, MK165457, MN182533, MK648157, MK133093, KT383474) representing outbreak locations in the Middle East, North-America and Europe.

In early October 2019, the Dutch National Plant Protection Organization (NPPO-NL) received a tomato sample as part of a broad generic survey for tomato pathogens, that was found to be infected with ToBRFV. After initial testing with DAS-ELISA, bio-assay and real-time RT-PCR, the sample was subjected to Illumina RNAseq analysis for final confirmation. Comparison of the (near) complete viral genome present in the sample with public NCBI accessions verified the presence of ToBRFV [17]. ToBRFV is regulated as part of EU Commission implementing decision 2019/1615 as of 1 November of 2019 [22]. Therefore, the initial detection triggered follow-up action targeting the nursery and the seed lots related to the infected plants, Additionally, intensified specific surveillance was carried out by the NPPO-NL to determine the extent of ToBRFV presence in the Dutch tomato horticulture. This surveillance resulted in new ToBRFV findings at tomato fruit companies and prompted the enforcement of measures to prevent further spread of the virus.

The aim of the current study was to determine the genomic diversity that was represented within and between the Dutch outbreak locations to identify possible linkages with epidemiological factors such as scion and rootstock varieties. We used Nextstrain [23], a collection of open-source bio-informatic tools, to create an interactive view of the diversity and spread of the virus. Real-time virus genome sequencing and rapid dissemination of epidemiologically relevant results, which is central in the Nextstrain philosophy, has been mainly applied to viruses causing disease in humans, such as Zika and Ebola [24]. By sharing the Dutch outbreak sequences in the context of geographical distribution and epidemiological traits when the outbreaks are still ongoing, will empower National Plant Protection Organizations (NPPOs) and the research community to better understand and control this damaging viral disease.

At the time of preparation of this report, public ToBRFV sequences and sequences generated in this study dating from November 2014 to November 2019 were included in the ToBRFV Nextstrain build. The Dutch NPPO (NPPO-NL) has committed to keep adding sequence data and improving the build. This report also serves as an invitation to other organizations to contribute their data to improve the predictive power of the tool. The ToBRFV Nextstrain build can be accessed via https://nextstrain.nrcnvwa.nl/ToBRFV/20191231 (last accessed: 30 July 2020).

## Material and methods

### Sampling and ToBRFV detection

Following the initial ToBRFV detection, companies were selected based on a risk assessment including potential linkages to the initial outbreak location and data collected from private laboratories. These companies were inspected by phytosanitary inspectors of NPPO-NL. From each company (which can consist of several greenhouses), at least one sample per greenhouse was taken. Typically, 50 young leaf parts (i.e. ripped leaf sections) were sampled from symptomatic tomato plants per greenhouse. When no symptomatic plants were found, asymptomatic material was sampled instead. In cases of reported ToBRFV suspicion but absence of tomato plants (e.g. as a result of crop rotation), residues from the previous crop such as tomato roots and/or water from rock wool were sampled. By November 2019, greenhouses of 68 companies were inspected, with a total of 135 samples collected for analysis. In addition to the survey samples taken at Dutch companies, two Egyptian fruit samples (39070022 and 39070030) intercepted from regular import inspections and found to be infected with ToBRFV were included in this study.

Glasshouse leaf samples containing 50 young leaf parts were split into two equal subsamples. From each subsample containing 25 leaf parts, a disc from each leaf was obtained using a 6 mm diameter leaf punch. The RNA from leaf discs of each subsample were extracted with the RNA extraction with the Sbeadex Maxi Plant Kit (LCG genomics, United Kingdom) on the automated KingFisher Flex 96 platform (ThermoFisher, MA, USA). Punch outs were added to 5 mL GH+ buffer [25] (6 M guanidine hydrochloride, 0.2 M sodium acetate pH 5.2, 25 mM EDTA, and 2.5% PVP-10) spiked with Bacopa chlorosis virus (BaCV) and homogenized with a Homex 6 (Bioreba, Switzerland) prior to RNA extraction. Extracted RNA was tested directly, or stored at -80 °C until use. Real-time RT-PCR reactions were performed based on the ISHI-VEG protocol for ToBRFV detection [26] to select RNA for Illumina sequencing. In short, reaction mixes contained two primer-probe pairs targeting the ToBRFV genome, and one targeting the BaCV spike-in (Table 1). Reaction mixes consisted 1x UltraPlex 1-Step ToughMix (Quanta Biosciences, MD, USA), 300 nM of each forward and reverse primer, 200 nM of each probe, and 3 μL of RNA template. Molecular grade water was added to reach a final volume of 25 μL. Real-time RT-PCR reactions were performed in a CFX96 thermal cycler (BioRad, CA, USA) using the following conditions: 50°C for 10 min, 95°C for 3 min, followed by 40 cycles of 95°C for 10 sec and 60°C for 1 min, and plate read at 60°C. When one or both ToBRFV primer-probe combinations produced a Cq value of ≤30, the virus was regarded to be detected. When both ToBRFV combinations failed to produce a Cq value ≤30 and the spike-in resulted in a Cq value of ≤32, the virus was not detected. When both ToBRFV combinations resulted in Cq values >30, and the spike-in resulted in a Cq value of >32, the result was undetermined, and testing was repeated. RNA extracts in which ToBRFV was detected were subjected to Illumina sequencing.

**Table 1:** Oligonucleotides described in the ToBRFV ISHI-Veg protocol [26] used in this study.

Name	Sequence 5’—3’	Target
CSP1325-F	CATTTGAAAGTGCATCCGGTTT	Coat protein (CP) and 3’ tRNA-like end
CSP1325-R	GTACCACGTGTGTTTGCAGACA	
CSP1325-P	FAM-ATGGTCCTCTGCACCTGCATCTTGAGA-BHQ1	
CaTa28-F	GGTGGTGTCAGTGTCTGTTT	Movement protein (MP)
CaTa28-R	GCGTCCTTGGTAGTGATGTT	
CaTa28-P	ATTO532-AGAGAATGGAGAGAGCGGACGAGG-BHQ1	
BaCV-F	CGATGGGAATTCACTTTCGT	Spike-in control
BaCV-R	AATCCACATCGCACACAAGA	
BaCV-P	TxR-CAATCCTCACATGATGAGATGCCG-BHQ2	

### Illumina sequencing

RNA extracts in which the virus was detected with the real-time RT-PCR (*N* = 70) were DNAse I treated (Qiagen, Germany) for 15 min at room temperature prior to sequencing. Samples were sent to GenomeScan (Leiden, the Netherlands) for generation of 2 Gb Illumina RNAseq 150PE (paired-end) data per sample. The Ultra II Directional RNA Library Prep Kit for Illumina (New England Biolabs, MA, USA) was used to process the samples according to the protocol "NEBNext Ultra II Directional RNA Library Prep Kit for Illumina". Briefly, rRNA was depleted from total RNA using the rRNA removal kit Ribo-Zero Plant (Illumina, CA, USA). After fragmentation of the rRNA reduced RNA, a cDNA synthesis was performed and used for ligation with the sequencing adapters and PCR amplification of the resulting product. Quality and yield after sample preparation were measured with a Fragment Analyzer (Agilent, CA, USA) prior to pooling for sequencing on an Illumina NovaSeq (Illumina, CA, USA). Subsample 39941596B was sequenced twice in independent sequencing runs to determine the repeatability of Illumina sequencing and the verification pipeline.

### Verification pipeline

RNAseq data were uploaded into CLC Genomics workbench v11.0.1 (Qiagen, Germany) and run in a custom workflow build for detection of *de novo* assembled viral contigs (S1 Fig). First, a quality trim (quality limit = 0.05; ambiguous limit = 2) was performed, followed by a *de novo* assembly (map reads back to contigs = on; length fraction = 0.8; similarity fraction = 0.8, minimum contig length = 200) and consensus sequences extraction (low coverage threshold = 10; remove regions with low coverage = on; post-remove action = join). The *de novo* assembled contigs were analyzed using the Basic Local Alignment Search Tool (BLAST, maximum alignments per database sequence = 5; maximum E-value = 1e-6, minimum identity = 70%) with a local installation of the NCBI nr/nt database [27]. Blast results were split into plant and non-plant hits, which were visualized in Krona (bit score threshold = 25) [28] (S2 Fig). The same pipeline was repeated with 1% of all reads as *de novo* assembly of high coverage contigs can be problematic resulting in fragmented assemblies. CLC external applications that were used in the pipeline are deposited on GitHub (https://github.com/NPPO-NL/CLC-External-Applications). Putative ToBRFV sequences were manually extracted from the assemblies, and reliability of the assembled putative ToBRFV contigs were determined by visually inspecting read mappings. Consensus sequences were annotated in Geneious R11 (Biomatters, New Zealand) based on the publicly available ToBRFV Tom1-Jo genome KT383474 [1] (minimal sequence similarity = 90%) and the Geneious ORF finder tool (genetic code = Table 1, minimum size = 200), and submitted in NCBI GenBank under accessions MN882011 to MN882064. The corresponding SRAs were submitted under accessions ERS4296142 to ERS4296201. The sequences generated in this study are (near) complete genome sequences. Due to technical limitations small parts of the 5’ and 3’ UTRs could have remained unassembled which is common when using Illumina sequencing.

In this study the term genotype is used to refer to any genomic diversity observed between (near) complete genome sequences. Frequency of major genotypes in the individual samples was determined with the “Basic Variant Calling” tool in CLC Genomics Workbench v11.0.1. (ploidy = 0, minimum coverage = 20, minimum count = 2, minimum frequency = 10%, base quality filter = default) using read-mappings to ToBRFV genome KX619418.

A phylogenetic tree was constructed with the ToBRFV genome sequences generated in this study, together with publicly available (near) complete ToBRFV genome sequences. Models for nucleotide substitution of the MAFFT [29] aligned sequences were obtained using the “model testing” tool in CLC Genomics workbench v11.0.1 which was run with default settings for the Hierarchical likelihood ratio test (hLRT), Bayesian information criterion (BIC), Minimum theoretical information criterion (AIC), and Minimum corrected theoretical information criterion (AICc). The clustering analysis was performed with MrBayes v3.2.6 [30] implemented in Geneious R11 under the GTR model with gamma distribution and estimation of invariable sites (GTR + G + I) with a random starting tree and four Monte Carlo Markov Chains for 10^6^ generations. Nucleotide positions with <100% coverage on the terminal ends of the alignment were masked and not included in the clustering analysis. Trees were sampled every 200 generations, and the first 10^5^ generations were discarded as burn-in. Remaining trees were combined to generate a 50% majority rule consensus tree with posterior probabilities.

### Nextstrain implementation

Nextstrain is a bioinformatics pipeline that uses two tools, Augur and Auspice [23]. Augur (github.com/Nextstrain/augur) is a bioinformatics toolkit for phylogenetic analysis that uses a series of modules that produces output that subsequently can be visualized by Auspice (github.com/Nextstrain/auspice). Using the augur modules, ToBRFV sequences were aligned with MAFFT [29] and a RAxML clustering [31] was performed. The initial tree was refined with metadata and a time tree was build using TreeTime [32] with optimization of scalar coalescent time. Internal nodes were assigned to their marginally most likely dates, and confidence intervals for node dates were estimated. Amino acid changes were determined based on the functionally annotated Tom1-Jo genome (KT383474 = NC_028478). Additional features had to be added to the NCBI Genbank file (.gb) to enable the use of this sequence as reference, i.e. gene names and translation tables for the four CDS annotations. Next, Augur output was exported and visualized in auspice. The ToBRFV Nextstrain build is deposited on GitHub (https://github.com/NPPO-NL/nextstrain-ToBRFV).

## Results

### ToBRFV detection

**Fig 1:**
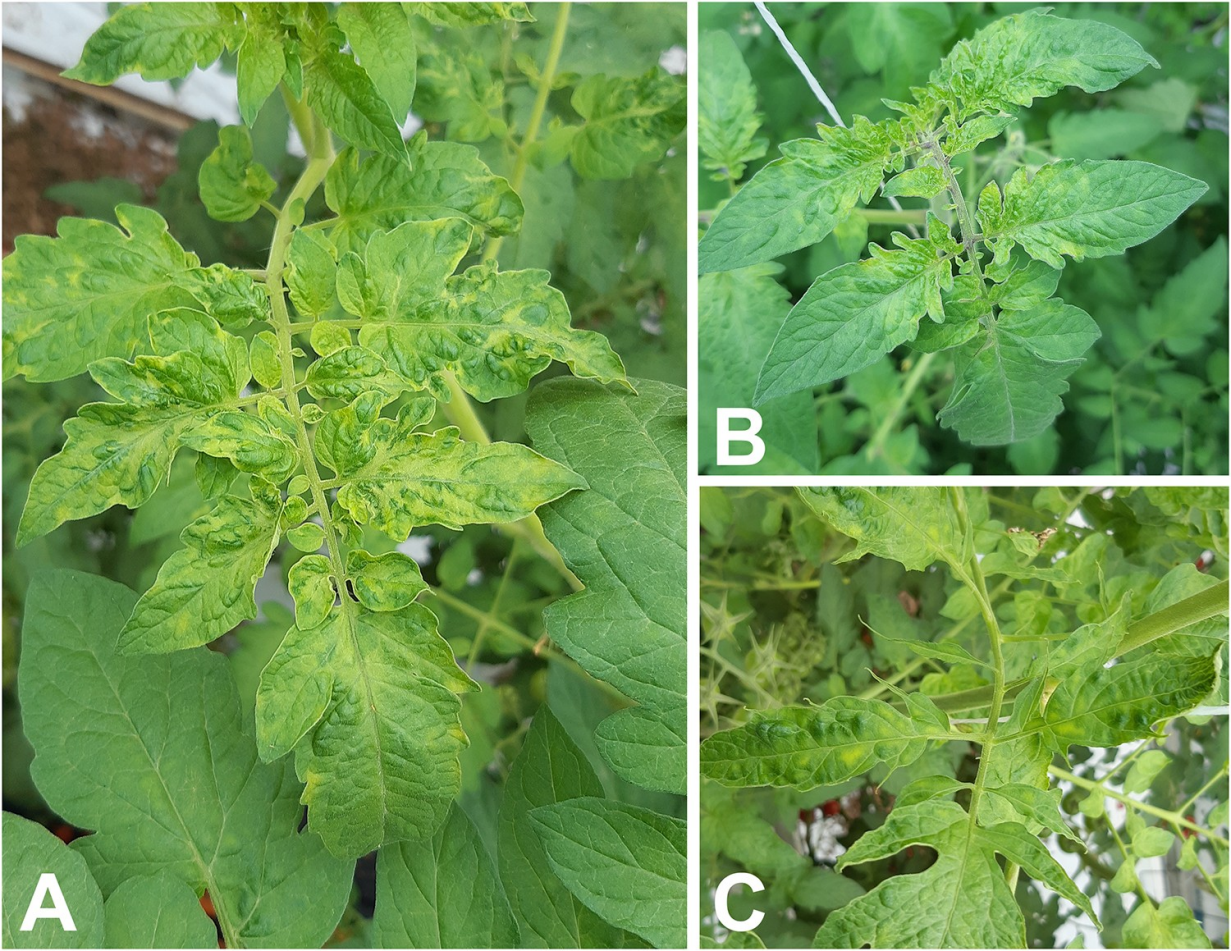
Symptoms observed during phytosanitary inspections. **A** Chlorotic mosaic on young leaves of ToBRFV and pepino mosaic virus infected tomato plants; **B** Chlorotic mottle on leaf bases of young leaves; and **C** narrowing of and chlorotic mottle on young leaves.

During the inspections that were part of the follow-up survey, young leaves with chlorotic mottle, and occasionally narrowing of leaves were observed. Additionally, narrowing of young leaves was occasionally observed (Fig 1). In three cases growers reported delayed ripening or brown necrotic spots on green fruits. Of the 135 samples taken during the follow-up survey, and the two fruit samples intercepted during import inspections, 70 samples (representing 17 companies and the two samples from import inspections) produced positive real-time RT-PCR results in one or both assays with Cq values ranging from 5.0 to 30.1 in the CP assay and from 4.5 to 31.2 in the MP assay. An overview of the samples that tested positive with the real-time RT-PCRs is provided in S1 Table.

### ToBRFV verification using Illumina RNAseq

On average 3.7 Gb of sequence data was generated per sample, and *de novo* assembly resulted in an average of 19,645 scaffolds per dataset when using all data. When the analysis with all data failed to produce a single contiguous sequence on account of too many reads that were used in the assembly, (near) complete genomes could be obtained with the 1% sampled analysis. Contigs producing blast hits for ToBRFV were obtained from datasets representing 17 companies and the fruits intercepted at import. Visual inspection of the *de novo* assemblies and structural annotation of these contigs verified the identity of the virus for all contigs producing ToBRFV hits. In two datasets obtained from sample 39941641, two different genotypes were assembled resulting in a total of 54 assembled and verified (near) complete ToBRFV genomes. The obtained genome size ranged from 6,301 to 6,397 nt with average read coverage ranging from 13 to 579,611x (median coverage = 54,330x). The subsample that was sequenced twice (i.e. 39941596_B) in independent sequencing runs as repeatability control produced two identical ToBRFV genome sequences (named 39941596 B-1 and 39941596 B-2 in the Nextstrain build). Both sequences represent a unique genotype in the overall dataset.

Subsamples in which ToBRFV could be verified typically had Cq values for the CP and MP assays lower than 25. Only in two cases the (near) complete ToBRFV genome could be constructed from samples with Cq values higher than 25. In some of the samples with Cq values higher than 25, low numbers of reads mapping to the ToBRFV could be obtained, but these were insufficient to verify the presence of ToBRFV in the sample. Apart from ToBRFV hits, RNAseq data obtained from the all sequenced tomato plants produced pepino mosaic virus (PepMV) hits.

### Genomic variation

The obtained ToBRFV sequences were highly similar with 99.3% to 100% identical sites (up to 43 single nucleotide polymorphisms: SNPs). For the 160 amino acid (aa) coat protein, no non-synonymous nucleotide changes were observed among the Dutch outbreak sequences, whereas the 1,621 aa RdRp protein, which contains the small replicase subunit, showed 100% to 99.8% similarity on protein level (up to 6 aa changes). The 267 aa movement protein displays relatively the highest level of diversity on protein level with 100% to 98.1% aa similarity (up to 5 aa changes).

**Fig 2:**
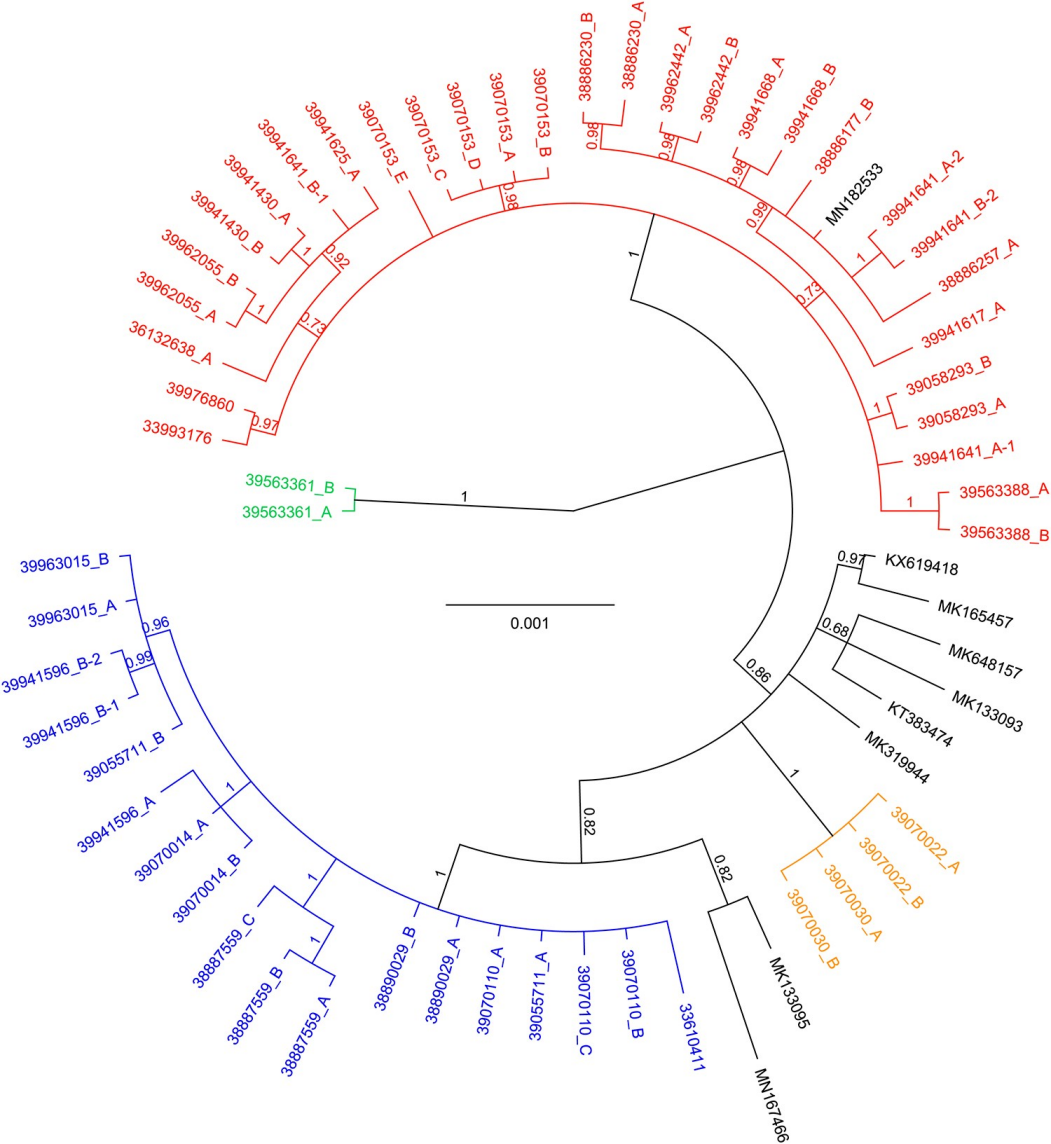
Bayesian inference of phylogeny based on a 6,404 nt alignment of 63 complete ToBRFV genomes. Viral strains from the Dutch outbreak are found in three separate clusters which have been colored red, blue and green. Four ToBRFV genome sequences generated from material intercepted during an import inspection from Egypt form a separate cluster (orange). Bayesian posterior probabilities (PP) are displayed at the branch nodes. The scale bar indicates the number of substitutions per site.

Bayesian inference of phylogeny results in clustering of the ToBRFV genomes sequenced from the Dutch outbreak locations in three main clusters (Fig 2: red, blue and green clusters). A fourth cluster was formed with sequences obtained from tomato fruits intercepted during import from Egypt (Fig 2: orange cluster). Clustering obtained with RAxML, which is part of the Nextstrain build, results in the same grouping as the Bayesian analysis for the viral strains obtained from the Dutch outbreak locations, but some rearrangements can be seen between clusters (S3 Fig).

**Fig 3:**
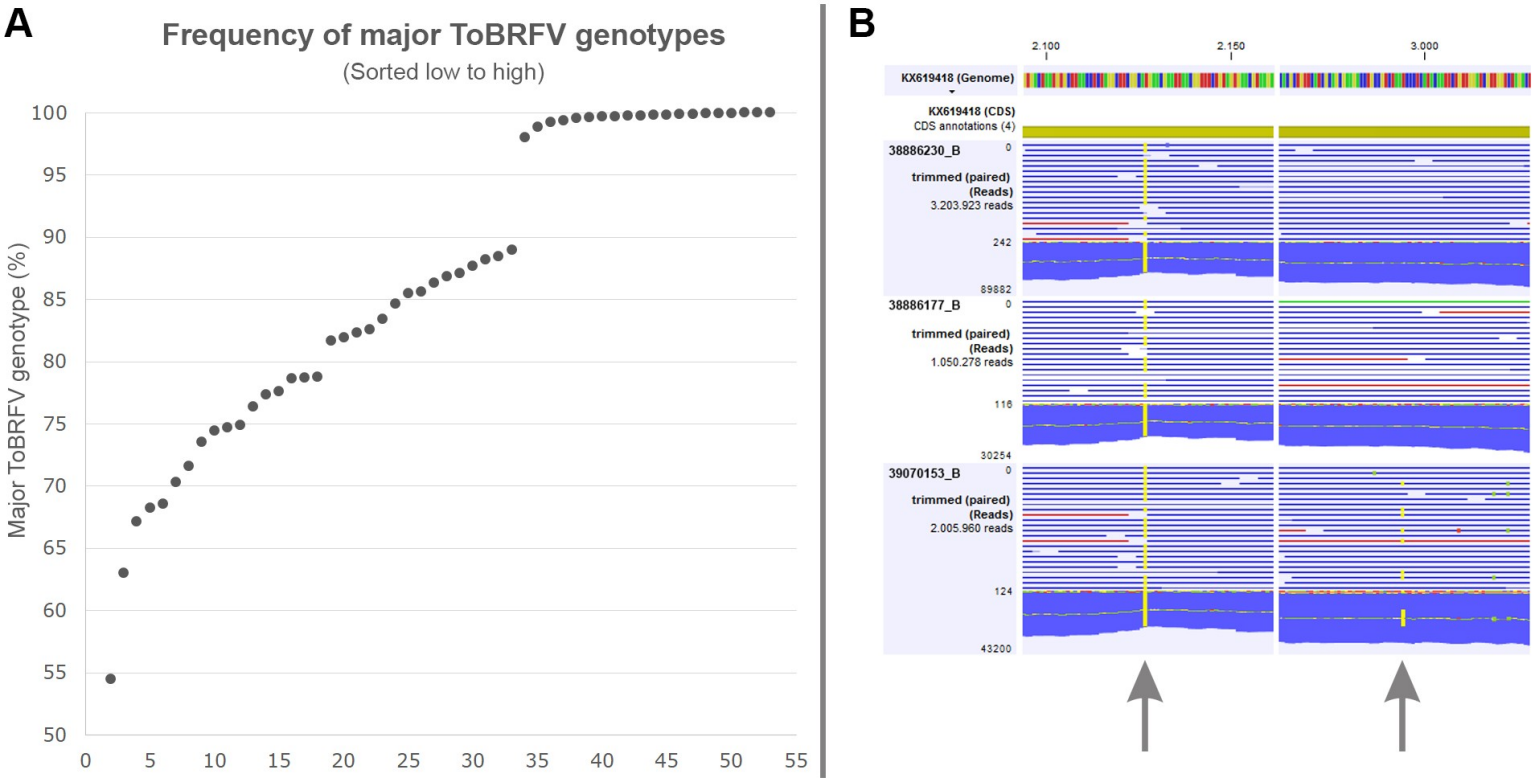
Within sample diversity of ToBRFV genotypes. **A**. Frequency (y-axis) of major ToBRFV genotypes in datasets (x-axis) generated in this study. Within sample frequencies of major genotypes are sorted low to high. **B**. Detail of RNAseq read mappings to the small replicase subunit of KX619418 for ToBRFV samples 38886230_B, 38886177_B, and 39070153_B. For each sample the total number of mapped reads is shown as well as the maximum read coverage. The left SNP (arrow) represents a single genotype (>99.7%) that is present in all three samples. The right SNP reveals the presence of a minor genotype (32.8%) in sample 39070153_B.

In twenty datasets, only a single ToBRFV genotype was detected (major genotype ≥98%), but in the remaining 34 datasets intermediate SNPs were observed suggesting the presence of multiple genotypes within the sample (Fig 3). Within sample diversity of major genotypes ranged from 54.5% to 89.0% in the isolates with mixed genotypes.

### Nextstrain build

The ToBRFV Nextstrain build contains 63 complete ToBRFV genomes generated from material sampled between November 2014 and November 2019. Information in the build is presented in three main panels: clustering of genomic diversity, geographical origin of the samples, and diversity relative to the ToBRFV Tom1-Jo genome (KT383474 = NC_028478).

The associated metadata included in the build allows users to color the nodes in the tree according to the host plant, and the (anonymized) rootstock and scion varieties from which the sequence was obtained. In addition, the within-sample genomic diversity is a trait that can be visualized. Internal node colors indicate the predicted ancestral state of a given trait, and the confidence of that state is conveyed by saturation of the color of the internal node. The cladogram can be shown in different styles such as rectangular, radial and unrooted. The branch lengths of the tree can be shown based on divergence or in function of time. Based on the information provided in the build, Nextstrain determines the most likely transmission events, which can be animated from the webpage. The genotypes represented in the tree are plotted on a map, and users can set different levels of geographical resolution, i.e. continent, country, state and municipality (when this information is available). This allows simultaneous interrogation of phylogenetic and geographic relationships, with additional relevant metadata. Users can download metadata and tree-files from the webpage, and can create screenshots of their views.

## Discussion

Understanding when viral outbreaks occur and which factors play a role in the introduction and spread are critical in defining control strategies and to prevent future outbreaks. Integrating epidemiological data with complete viral genomes has been applied in the public health domain to unravel transmission dynamics, including local transmission and global spread [reviewed by 33, 34]. The 2014–2015 Ebola outbreak is one of the first examples where complete viral genomes were used in conjunction with epidemiological data to achieve real-time tracking of the disease outbreak [35]. The public Nextstrain tool has been instrumental in visualizing the genomic data in the context of geographic distribution and multi-layered epidemiological traits for several viral and bacterial human pathogens including the novel coronavirus causing the ongoing COVID-19 pandemic (https://nextstrain.org/ncov/global, last accessed 24-7-2020). In the plant health domain, two Nextstrain builds are available. One for the wheat yellow rust pathogen *Puccinia striiformis* f. sp. *tritici* [36], and one for cassava-infecting geminiviruses in Africa (https://nextstrain.org/community/pestdisplace/CMDAFRICA, last accessed 24-7-2020). To our knowledge, this paper represents the first example in plant health where (near) complete pathogen genomes are used in real-time tracking of disease outbreaks.

In this study, presence of ToBRFV was verified with analysis of plant rRNA depleted Illumina RNA sequence data. An Illumina sequencing approach was preferred over Sanger sequence based verification even though primer pairs targeting different loci have been described and used for Sanger sequence based ToBRFV verification [2, 3, 10, 11, 13, 16, 37]. Public sequences obtained with Sanger sequencing cover either the 5’ end of the small replicase subunit, the 3’ end of the small replicase subunit, the 3’ end of RdRp, the partial MP or partial CP (S4 Fig). This inevitably results in fragmented and incomplete datasets which hampers harmonization of diagnostic protocols and the re-usability of sequence data in follow-up studies. In contrast to Sanger sequencing, RNAseq analysis with plant rRNA depleted Illumina data does not rely on the specificity of primers used to amplify the partial virus genome. With the data obtained with Illumina sequencing, the (near) complete genome can be used to determine potential linkages between genotypes and epidemiological traits.

### Pathogen detection and sequencing

The detection test used in this study was not part of the primary focus of our research. Nevertheless, we included details of the test protocol to present our Illumina sequence-based analyses in the context of real-time RT-PCR data. We used the ISHI-Veg protocol for pathogen detection as it is a multiplex assay that targets both the movement protein and coat protein genes allowing pathogen detection using two independent loci in a single reaction. In comparison, the real-time RT-PCR test as described by Panno *et al*. consists of a simplex assay targeting the movement protein gene [38]. ISHI-Veg is a standard setting body under the International Seed Federation (ISF) and ISHI-Veg methods are developed using the collective expertise and experience of its members. The validation of the ISHI-Veg protocol was performed for detection of ToBRFV in tomato and pepper seeds [26], and currently the ISHI-Veg protocol is further validated and compared to other serological and molecular tests in an interlaboratory test performance study under the European Valitest project [39]. When implementing the ISHI-Veg test for tomato leaves, late Cq values were obtained with the healthy tomato matrix. To differentiate between background signals and ToBRFV derived Cq values, a Cq cut-off value of 30 was introduced. RNA extracts producing Cq values lower than 25 could be verified with Illumina RNAseq data, and when Cq-values between 25 and 30 were obtained (*N* = 19), ToBRFV could be verified once. Overall there is a good correlation between the Cq values and normalized read coverage in the samples analyzed (*R*^*2*^_CP assay_ = 85.3% and *R*^*2*^_MP assay_ = 76.4%; S5 Fig).

Visual inspection of the read mappings revealed that multiple genotypes existed within some samples (i.e. the presence of different virus genotypes in a field population). Only in two subsamples the variation between the genotypes within a single subsample was high enough to produce two separate contigs from the *de novo* assembly. In other cases, the minor genotypes were not manually reconstructed from the assemblies as they could represent one or more than one minor genotypes. When regarding the within sample diversity, a gap of almost 10% between samples with one clear major genotype and samples with mixed genotypes was observed (Fig 3). Since reads were mapped to reference KX619418, variants mapping between 89.0 and 98.0% would have been detected if they were present. ToBRFV populations with only a single major genotype could have undergone a recent genetic bottleneck event. However, no correlation between within sample genomic diversity and any of epidemiological traits analyzed was observed that could explain such event.

### Epidemiology of Dutch ToBRFV outbreak

The ToBRFV Nextstrain build contains 63 (near) complete ToBRFV genomes, 54 of which have been generated in this study. The genomic sequences generated from the Dutch outbreak sites group in three main clusters (Fig 2: red, blue, and green clusters). The TimeTree analysis estimates the divergence of the red and blue clusters well before the first finding of ToBRFV in the Netherlands (inferred date = May 2014, confidence interval = March 1980 –October 2014), and these groups are believed to have emerged before introduction into the Netherlands. Therefore, the three clusters are hypothesized to represent three original different sources. The blue and green clusters consist of sequences that so far were only found in the Netherlands. One sequence obtained from an outbreak in the United Kingdom groups in the red cluster, suggesting that these are potentially linked. In all but one of the inspected companies, ToBRFV genotypes obtained from the samples taken belonged to one of the main clusters. In a single company however, ToBRFV genotypes from both the red and blue cluster were obtained. These two different genotypes were obtained from separate greenhouses of the same company, and it is likely that they were introduced independently.

A number of epidemiological traits were analyzed in context of genomic diversity to identify the possible source(s) of the ToBRFV outbreak in the Netherlands. These included rootstock and scion varieties, young plant providers, seed batches and seed providers. However, no linkages were identified between these epidemiological traits and companies or genotypes. Given the distribution of epidemiological traits among genotypes and outbreak locations, it is likely that the virus has been present in the Netherlands some time before the first official samples were obtained and tested. Establishing the origin of tobamoviruses could also prove to be challenging as they are easily transmitted and can spread rapidly over short periods of time when they remain unnoticed. Companies that have been found to have outbreaks must take strict hygiene measures to prevent spread of the virus to other companies and to eradicate the virus at the end of the cropping season. Should the virus re-occur after crop rotation and intensive cleaning, the (near) complete genome sequences may prove instrumental in determining if a new infection was introduced from a new or existing source.

Apart from the genomic diversity of sequences obtained from the Dutch outbreak locations, genotypes found at outbreak locations in the Middle East, North-America, Italy and Germany form additional clusters. Genotypes from these other outbreak locations were not found in Dutch greenhouses. The genotype that was obtained from two Egyptian samples taken during an import inspection, represented a new genotype which previously had not been sequenced. Diversity is expected to be highest at the center of origin, and increased sampling at outbreak locations and presumed origin will aid further understanding of ToBRFV diversity. This will eventually allow the determination of the origin for this viral species.

The ToBRFV Nextstrain build currently holds ToBRFV genomes from various outbreak locations in the Middle East, North-America and Europe. However, there is a strong bias towards genotypes obtained from the Dutch outbreak locations. Sampling bias and lack of data can (in addition to the limited genomic variation and ease of transmission) hamper the determination of reliable transmission links. The predictive power of the tool will improve with the addition of additional genomes. Therefore, we encourage other organizations to share data or biological materials together with relevant metadata in order to improve the build. Such a community effort will contribute to source attribution and to a better understanding of the diversity and spread of ToBRFV worldwide. The ToBRFV Nextstrain build will be maintained by NPPO-NL.

## Supporting information

S1 Fig Visualization of the CLC Genomics workbench pipeline used to identify putative ToBRFV contigs. The pipeline combines a quality trim for input reads, *de novo* assembly, blast based detection and visualization of blast output in Krona. The pipeline runs on all input data, and on a random sample of 1% of all reads. Blue process steps indicate output, and custom plugins created for the pipeline are named CEA (CLC external application).
(TIF)

S2 Fig Example of Krona visualization of blast output for sample 38886230_A. When using all data for the *de novo* assembly, and after blast-based filtering of plant contigs, 1266 contigs remained. The majority of these sequences (99%) did not produce a blast hit, but 3 viral blast hits were obtained (left panel). When selecting the viral hits, the blast-based identity is shown (right panel). In this example, one ToBRFV hit and two PepMV hits are obtained. The sequence producing the ToBRFV hit is obtained via a link, which is then further analyzed to determine the presence of the virus in the sample.
(TIF)

S3 Fig Comparison of the Nextstrain RAxML tree (left) and the Bayesian inference of phylogeny (right) of 63 ToBRFV genomes included in this study. Links are drawn for the isolates generated in this study. The Nextstrain tree is colored based on the country of origin, whereas the Bayesian tree is colored based on the main clusters identified for the viral genomes sequenced in this study. Grouping within the main clusters is identical between the two analyses, but some groups are placed at different positions in the overall phylogeny (e.g. the Egyptian sequences: orange cluster).
(TIF)

S4 Fig Publicly available ToBRFV sequences mapped to ToBRFV genome NC_028478 (= KT383474). Coding sequences are annotated in yellow, and sequences identical to the reference sequence are shown in grey. Differences relative to the reference are highlighted in black. Apart from the nine (near) complete genome sequences, 37 Sanger sequences have been submitted to NCBI covering ① 5’ end of the small replicase subunit, ② 3’ end of the small replicase subunit, ③ 3’ end of RdRp, and ④ the partial MP and CP.
(TIF)

S5 Fig Correlation between Cq values and read normalized read mapping. Real-time RT-PCR Cq values of the CP (top) and MP (bottom) assays are displayed on the x-axis and the normalized average read-coverage is shown on the y-axis. To allow comparison between the different datasets, the average read coverage of the ToBRFV genome for a given sample was multiplied by the fraction of reads generated for that sample relative to the mean of reads generated for all samples.
(TIF)

S1 Table Details of real-time RT-PCR detection and RNAseq-based verification tests performed on ToBRFV samples included in this study. (XLSX)
