# Supplementary figures and images for: Real-time tracking of Tomato brown rugose fruit virus (ToBRFV) outbreaks in the Netherlands using Nextstrain
Source: PLoS One. 2020 Oct 8;15(10):e0234671. doi: 10.1371/journal.pone.0234671 (PMC7544112; doi:10.1371/journal.pone.0234671)

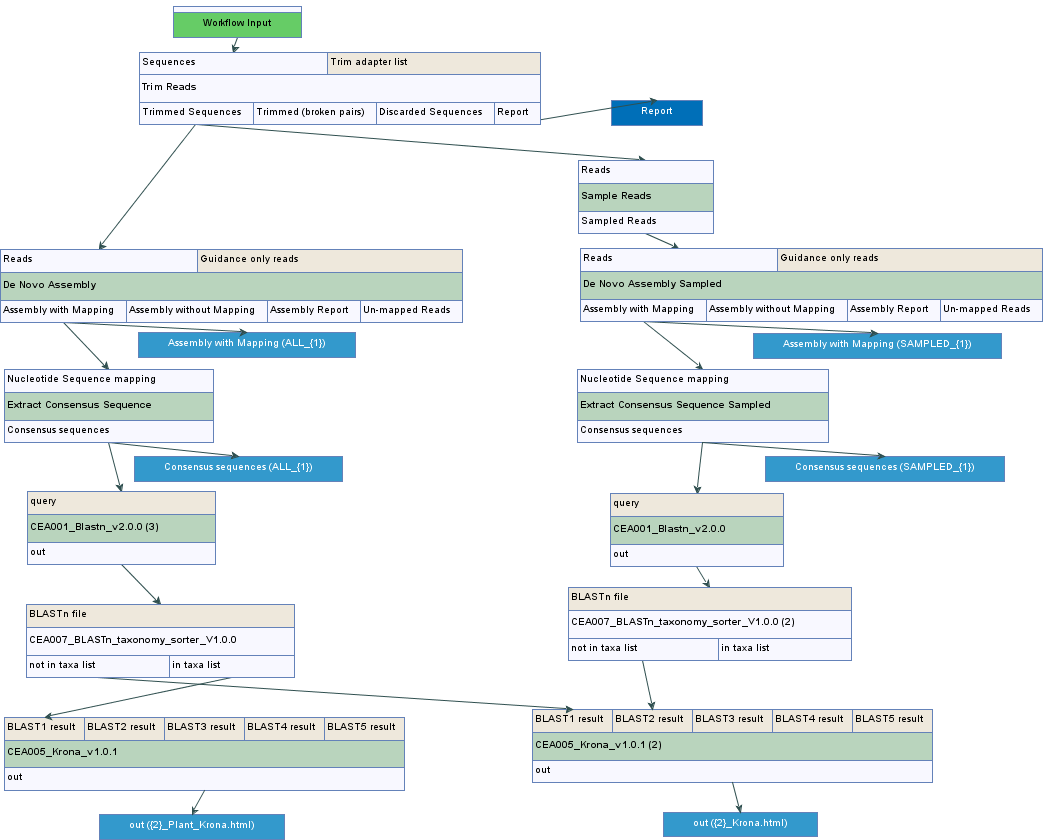

Supplement: S1 Fig — The pipeline combines a quality trim for input reads, de novo assembly, blast based detection and visualization of blast output in Krona. The pipeline runs on all input data, and on a random sample of 1% of all reads. Blue process steps indicate output, and custom plugins created for the pipeline are named CEA (CLC external application). (TIF) [file pone.0234671.s001.tif]

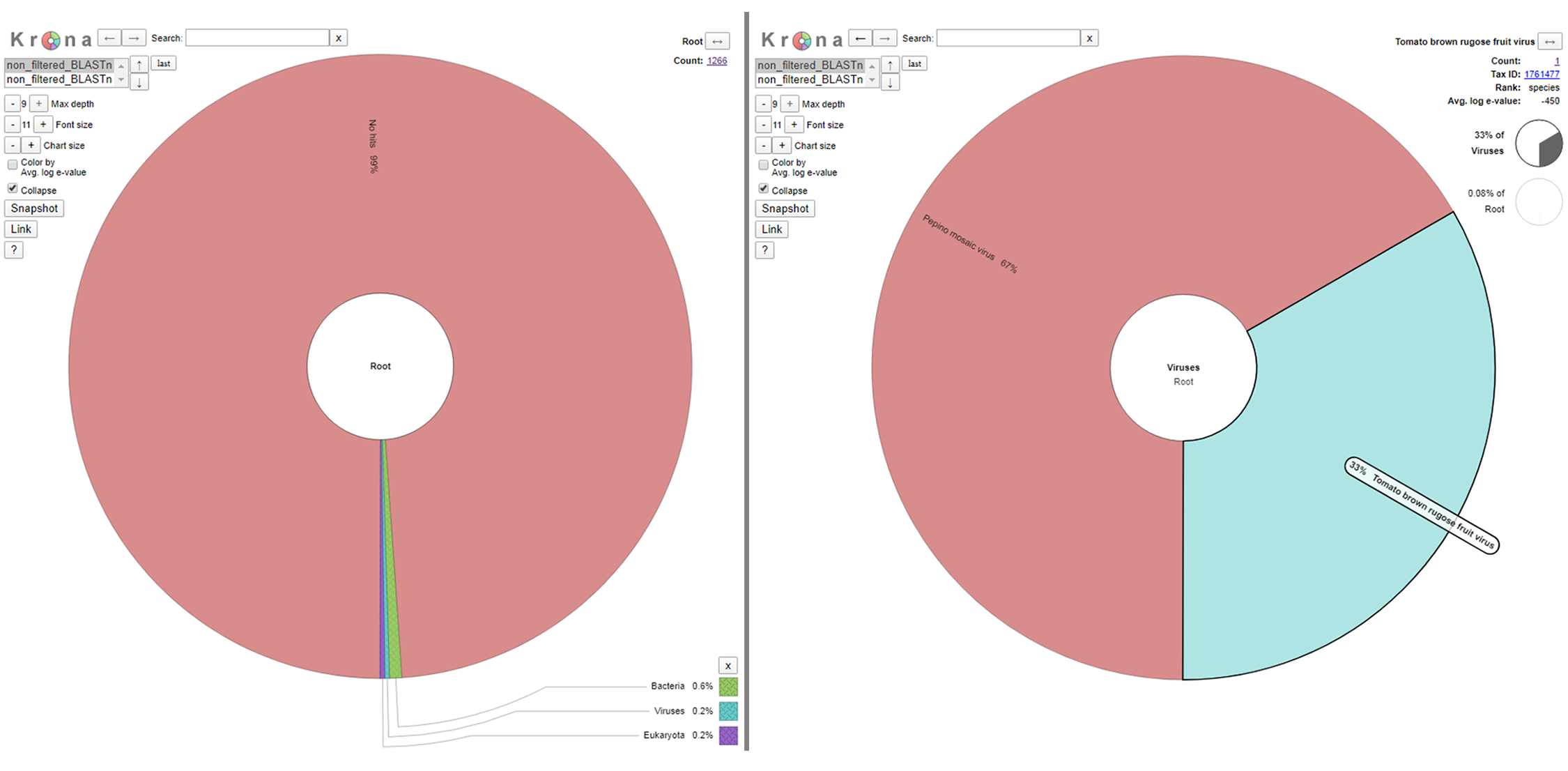

Supplement: S2 Fig — When using all data for the de novo assembly, and after blast-based filtering of plant contigs, 1266 contigs remained. The majority of these sequences (99%) did not produce a blast hit, but 3 viral blast hits were obtained (left panel). When selecting the viral hits, the blast-based identity is shown (right panel). In this example, one ToBRFV hit and two PepMV hits are obtained. The sequence producing the ToBRFV hit is obtained via a link, which is then further analyzed to determine the presence of the virus in the sample. (TIF) [file pone.0234671.s002.tif]

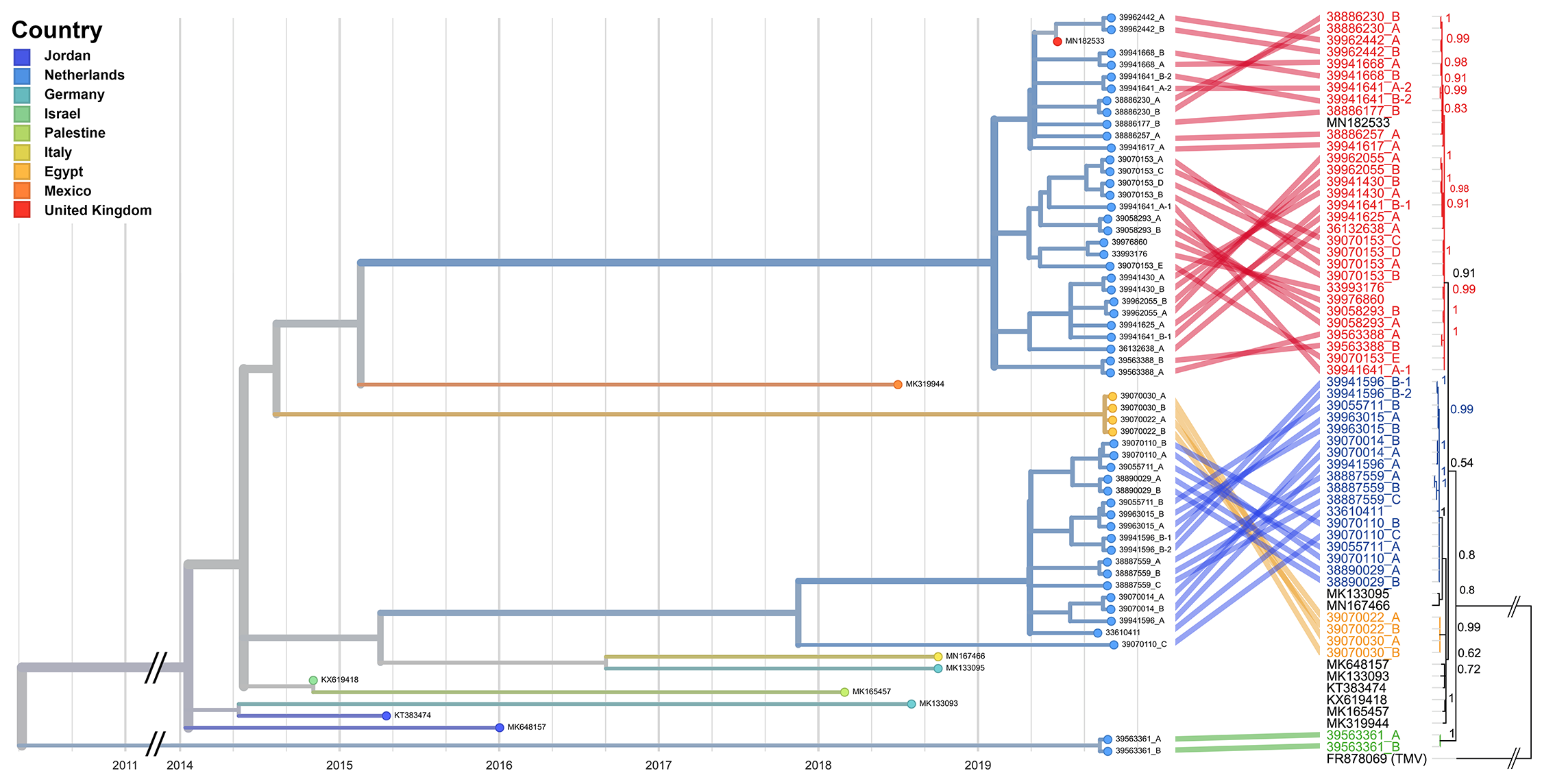

Supplement: S3 Fig — Links are drawn for the isolates generated in this study. The Nextstrain tree is colored based on the country of origin, whereas the Bayesian tree is colored based on the main clusters identified for the viral genomes sequenced in this study. Grouping within the main clusters is identical between the two analyses, but some groups are placed at different positions in the overall phylogeny (e.g. the Egyptian sequences: orange cluster). (TIF) [file pone.0234671.s003.tif]

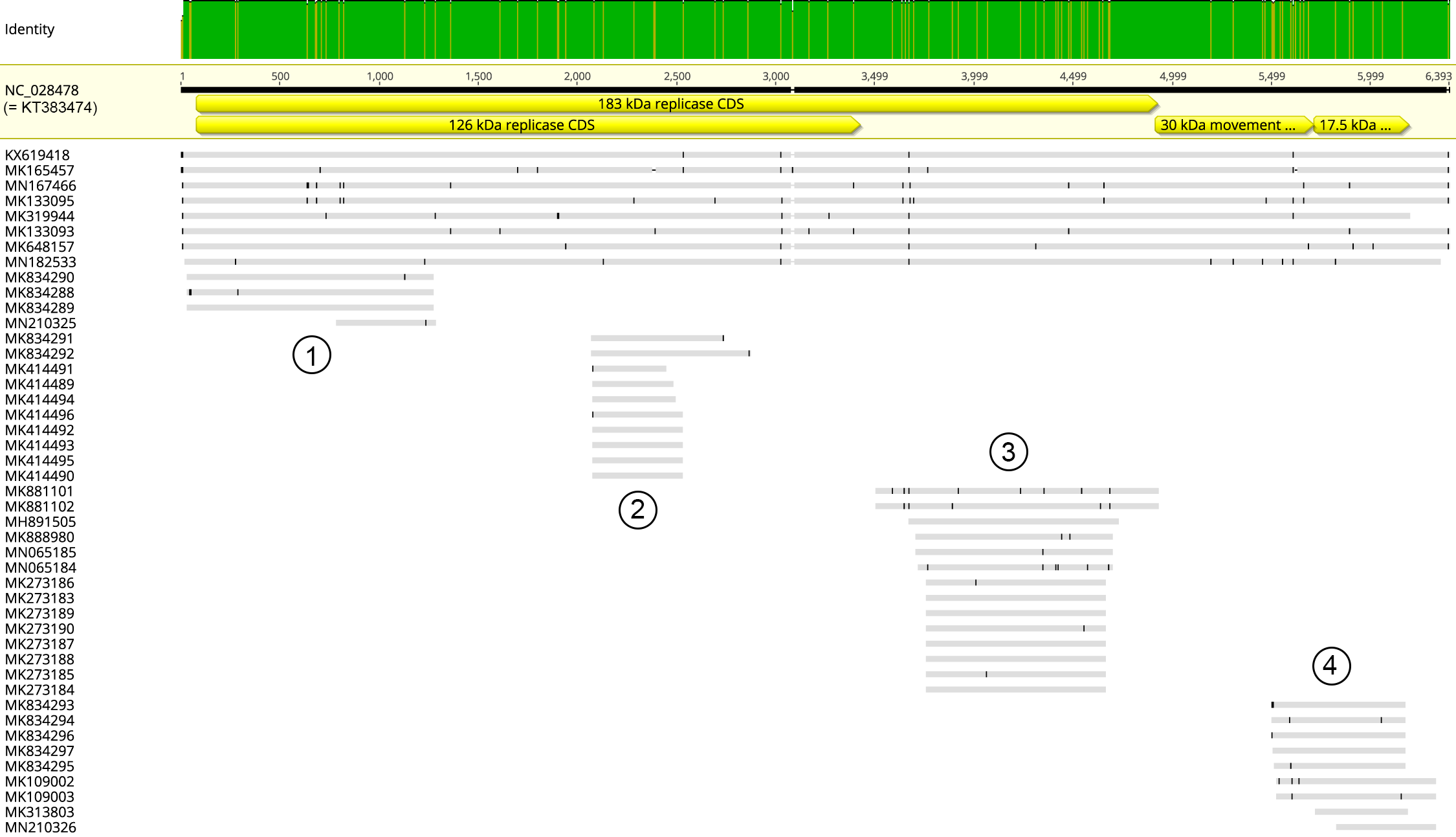

Supplement: S4 Fig — Coding sequences are annotated in yellow, and sequences identical to the reference sequence are shown in grey. Differences relative to the reference are highlighted in black. Apart from the nine (near) complete genome sequences, 37 Sanger sequences have been submitted to NCBI covering ① 5’ end of the small replicase subunit, ② 3’ end of the small replicase subunit, ③ 3’ end of RdRp, and ④ the partial MP and CP. (TIF) [file pone.0234671.s004.tif]

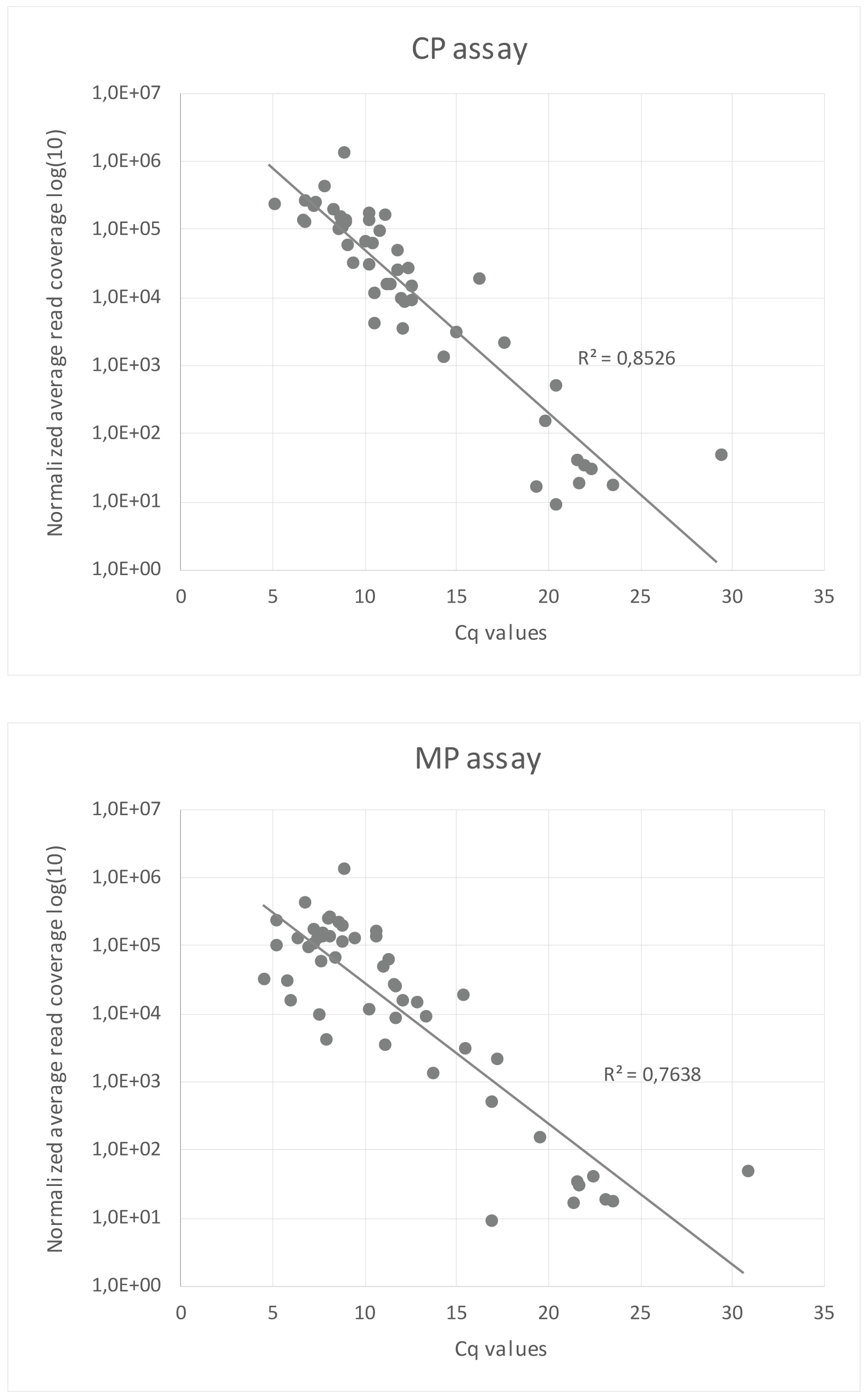

Supplement: S5 Fig — Real-time RT-PCR Cq values of the CP (top) and MP (bottom) assays are displayed on the x-axis and the normalized average read-coverage is shown on the y-axis. To allow comparison between the different datasets, the average read coverage of the ToBRFV genome for a given sample was multiplied by the fraction of reads generated for that sample relative to the mean of reads generated for all samples. (TIF) [file pone.0234671.s005.tif]
